# Identification of *matB* used as an endogenous reference gene for the qualitative and real‐time quantitative polymerase chain reaction detection of *Lentinus edodes*

**DOI:** 10.1002/fsn3.2860

**Published:** 2022-04-01

**Authors:** Ling Li, Yuanmiao Wei, Yao Liu, Shuna Xiang, Hanyue Zhang, Ying Shang

**Affiliations:** ^1^ 47910 Faculty of Food Science and Engineering Kunming University of Science and Technology Kunming China

**Keywords:** endogenous reference gene, *Lentinus edodes*, *matB*, qualitative PCR, quantitative PCR

## Abstract

*Lentinus edodes* is a fungus with rich nutritional value and good medicinal value and has accordingly become a substitute for other expensive wild edible mushrooms. In this study, for the first time, the *matB* gene was selected as an endogenous reference gene of *L. edodes* and identified as the species‐specific gene. The *matB* genes of *L. edodes* and 18 non‐*L. edodes* species were determined by qualitative polymerase chain reaction (PCR), but no amplification was found in non‐*L. edodes* species. In SYBR Green quantitative PCR analysis, the detection limit was as low as 16 pg/µl of DNA template. All of these experiments indicated that the *matB* gene is an ideal reference gene and can detect *L. edodes* material through qualitative and quantitative PCR assays. It also provides a convenient and accurate approach for the detection of *L. edodes* products and the adulteration in wild edible mushroom products.

## Graphical abstract

The *matB* gene can be used as an ideal endogenous reference gene for *L. edodes* in the PCR‐based detection, with a detection limit of DNA as low as 16 pg/µl in SYBR Green quantitative PCR analysis. This method can be used not only for *L. edodes* raw materials but also for *L. edodes* products or other types of processed mushroom foods.

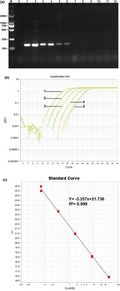

## 1 INTRODUCTION

*Lentinus edodes* belongs to class Agaricomycetes, subclass Agaricomycetidae, order Agaricales, family Omphalotaceae, and has been cultivated in China and Japan for more than 2000 years (Sheng et al., 2021). It is one of the second‐largest cultivated medicinal and edible mushrooms in the world, accounting for approximately 25% of the world's mushroom production (Bisen et al., 2010; Lee et al., 2019). *L. edodes* has satisfying flavor, nutritional characteristics, and medicinal value. It is low in lipids, high in protein and fiber, and contains various vitamins and minerals (Aly Farag El Sheikha & Hu, 2018; Gaitan‐Hernandez et al., 2019). It also contains lentinan, eritadenine, and other bioactive compounds, with anticancer, immunity‐improving, and antitumor activities (Shimada et al., 2003; Y. Y. Zhang et al., 2011) that can be processed into all kinds of nutritious and delicious food. In addition, some mushrooms also have biocompounds with insecticidal activity that are potentially useful as promising and powerful biopesticides (A. F. El Sheikha, 2021). However, with the development and progress of technology and food processing, food adulteration may occur, such as disguising *L. edodes* as other expensive wild edible mushrooms used in processed food. Therefore, to prevent this adulteration, a method of identifying *L. edodes* is necessary to develop.

Traditional detection methods include morphological and physiochemical identification. Morphological identification is macroscopic or morphological observation based on the experience of the shape, color, secretion, and odor of each part of the fruiting body (e.g., pileus, stipe, lamella, and volva) (Sugawara et al., 2016), there are artificial identification method and electronic nose technology (Wei et al., 2022). However, processed products have to undergo complex processes such as crushing, mixing, and chemical reactions. As a result, their morphological characteristics become too damaged to be used for identification. Physiochemical identification is based on the analysis of the chemical composition of food samples for identification (Danezis et al., 2016). Gas chromatography (Carvalho et al., 2014), liquid chromatography (Xu et al., 2017), mass spectrometry (Ogrinc et al., 2003), spectroscopy (Ma et al., 2020), nuclear magnetic resonance (Kuballa et al., 2018), and other techniques have been applied to detect food adulteration.

With the progress of modern biotechnology, molecular biology methods have been extensively used in the field of food authenticity identification (Ali et al., 2014; Farag et al.). DNA carries the genetic material of life and is extremely stable (Hong et al., 2017). Thus, it is very suitable for the detection and analysis of raw material components of deep processed products. DNA‐based food species identification methods rely mostly on the polymerase chain reaction (PCR) amplification of highly specific DNA fragments in the genome for detection purposes. This method is characterized by speediness, simplicity, specificity, and sensitivity (Bohme et al., 2019; Mafra et al., 2008). Other technologies include single‐strand conformation polymorphisms, random amplified polymorphic DNA, simple sequence repeat (SSR), inter‐SSR, DNA amplification fingerprinting, ribosomal DNA (rDNA) internal transcribed spacer (ITS) (Danezis et al., 2016; Das et al., 2013). However, these technologies either require the design of multiple pairs of primers for amplification or the later amplification products need to be sequenced through sequence alignment to achieve the purpose of detection.

In contrast, the endogenous‐based detection technique is more convenient and economical, which is amplified only by ordinary PCR, and the amplified product does not need to be sequenced. Endogenous reference genes refer to conserved DNA sequences with a single or constant low copy number and low variation that are species specific (Xue et al., 2014). The endogenous reference gene of a specific species is a genetic marker to distinguish other species and can be used in many fields, such as species and product identification (L. Zhang et al., 2009), product adulteration identification (Xiang et al., 2017), and transgenic component detection (Huang et al., 2013). Many applications of endogenous reference genes in different species have been reported, such as the *Lhcb2* gene in peach (Shang et al., 2014), the *Sad1* gene in transgenic cotton (Yang et al., 2005), the *RPL21* gene in wheat (Liu et al., 2014), the *LAT52* in tomato (Yang et al., 2008), the *SPS*, *GOS9*, *PLD*, and *ppi‐PPF* gene in rice (Wang et al., 2010), the *CTOS* gene in *Carthamus tinctorius* (L. Zhang et al., 2017), the *pol* gene in *Tricholoma matsutake* (Shan et al., 2019), and the *pyrG* gene in *Pleurotus ostreatus* (Zheng et al., 2018). However, no study has reported on the application of endogenous reference genes in the detection of *L. edodes* or the adulteration of wild mushroom products.

In the detection of food adulteration, the PCR amplification of endogenous reference gene is becoming increasingly important. In the present study, the *matB* gene was selected as an endogenous reference gene of *L. edodes* by intra‐ and interspecies identification. The specificity and detection limit of *matB* gene was verified by qualitative and real‐time quantitative PCR. This study aimed to establish a convenient and accurate method for the detection of *L. edodes* products and the detection of adulteration in wild edible mushrooms products.

## 2 MATERIALS AND METHODS

### 2.1 Materials

Nineteen types of fresh mushrooms were purchased from local markets in Kunming city, Yunnan Province. The mushroom species were first identified by professionals through the artificial identification method and reverified by specific primers during the experiment (Aly Farag El Sheikha & Hu, 2018; Wei et al., 2022). The mushrooms included *Lentinula edodes* and 18 other mushrooms, namely, *Boletus auripes*, *Pleurotus ostreatus*, *Boletus brunneissimus*, *Lactarius volemus*, *Russula virescens*, *Thelephora ganbajun*, *Cantharellus cibarius*, *Tricholoma matsutake*, *Boletus rubellus*, *Boletus griseus*, *Russula vinosa*, *Boletus subsplendidus*, *Termitornyces albuminosus*, *Catathelasma ventricosum*, *Ramaria botrytoides*, *Agrocybe aegerita*, *Pleurotus eryngii*, and *Tricholoma gambosum*.

### 2.2 Oligonucleotide primers and dye

All primers were designed with ABI Prism Primer Express version 3.0 (Applied Biosystems) and synthesized by Shanghai Sangon Co., Ltd. The fungus universal primer ITS rDNA‐1F/4R DNA was used to assess the genome quality. The primers *matB*‐F/R and SYBR Green (Tiangen Biotech Co., Ltd.) were used to detect the *matB* gene of *L. edodes* through qualitative and real‐time quantitative PCR. The detailed nucleotide sequences of the primers are listed in Table 1.

**TABLE 1:** Primers used in qualitative and quantitative PCR

Primer name	Primer sequence (5′ → 3′)	Length	Product size (bp)	Reference
ITS1‐F	TCCGTAGGTGAACCTGCGG	19	675	This study
ITS−4R	TCCTCCGCTTATTGATATGC	20		
*matB*‐F	AACGAGGTTGAGGATCGGG	19	142	
*matB*‐R	CAATTGAAGGGAAGTGAGTGGT	22		

### 2.3 Extraction of genome and verification

The total genome of all mushrooms was extracted using kit method (Ezup Column Fungi Genomic DNA Purification Kit, B518259; Sangon Biotech Co., Ltd., Shanghai, China). DNA concentration and purity were determined and calculated using NanoDrop 2000 (Thermo Fisher Scientific Inc). Genomic DNA integrity was analyzed by 1% agarose gel electrophoresis with TS‐GelRed (TsingKe Biotech Co., Ltd.) in 1×TAE. The PCR products amplified by the fungus universal primer ITS were analyzed on 2% agarose gel.

### 2.4 PCR conditions

For the qualitative confirmation of species specificity, PCR reaction was performed at a total volume of 25 μl on an ABI SimpliAmp Thermal Cycler (Applied Biosystems, USA). Each reaction contained 2.5 μl of 10× buffer, 2 μl of dNTP (2.5 mM), 1 μl of each primer (10 µM), 2.5 units of Taq DNA polymerase (TaKaRa Biotechnology Co., Ltd.), and 2 μl of DNA template (Shan et al., 2019).

For ITS amplification, the PCR conditions were 5 min predenaturation at 95°C; 30 cycles of 30 s at 95°C, 30 s at 58°C, 30 s at 72°C; and termination of extension 5 min at 72°C (Zheng et al., 2018). The PCR system of *matB* gene was the same as that of ITS, except that the annealing temperature was 60°C. All PCR products were analyzed by 2% agarose gel electrophoresis with TS‐GelRed (TsingKe Biotech Co., Ltd.) in 1×TAE.

For quantitative sensitivity detection, SYBR Green real‐time quantitative PCR was used for amplification detection, and the total reaction system was 25 μl. Each reaction contained 12.5 μl of 2.5×SYBR Green Mix, 2.5 μl of 50×ROX Reference Dye (Tiangen Biotech Co., Ltd., Beijing, China), 0.75 μl of each primer (10 µM), and 2 μl of the DNA template (fivefold diluted). Real‐time PCR reactions were performed on an ABI Step One Plus detection system (Applied Biosystems, Foster City, USA) with the following program: 15 min at 95°C; 40 cycles of 10 s at 95°C, 30 s at 60°C, 30 s at 72°C. Then, the melting curve was analyzed. Each sample was quantified in duplicate, and three biological replicates were conducted.

## 3 RESULTS AND DISCUSSION

### 3.1 Selection of endogenous reference gene for *L. edodes*

**FIGURE 1:**
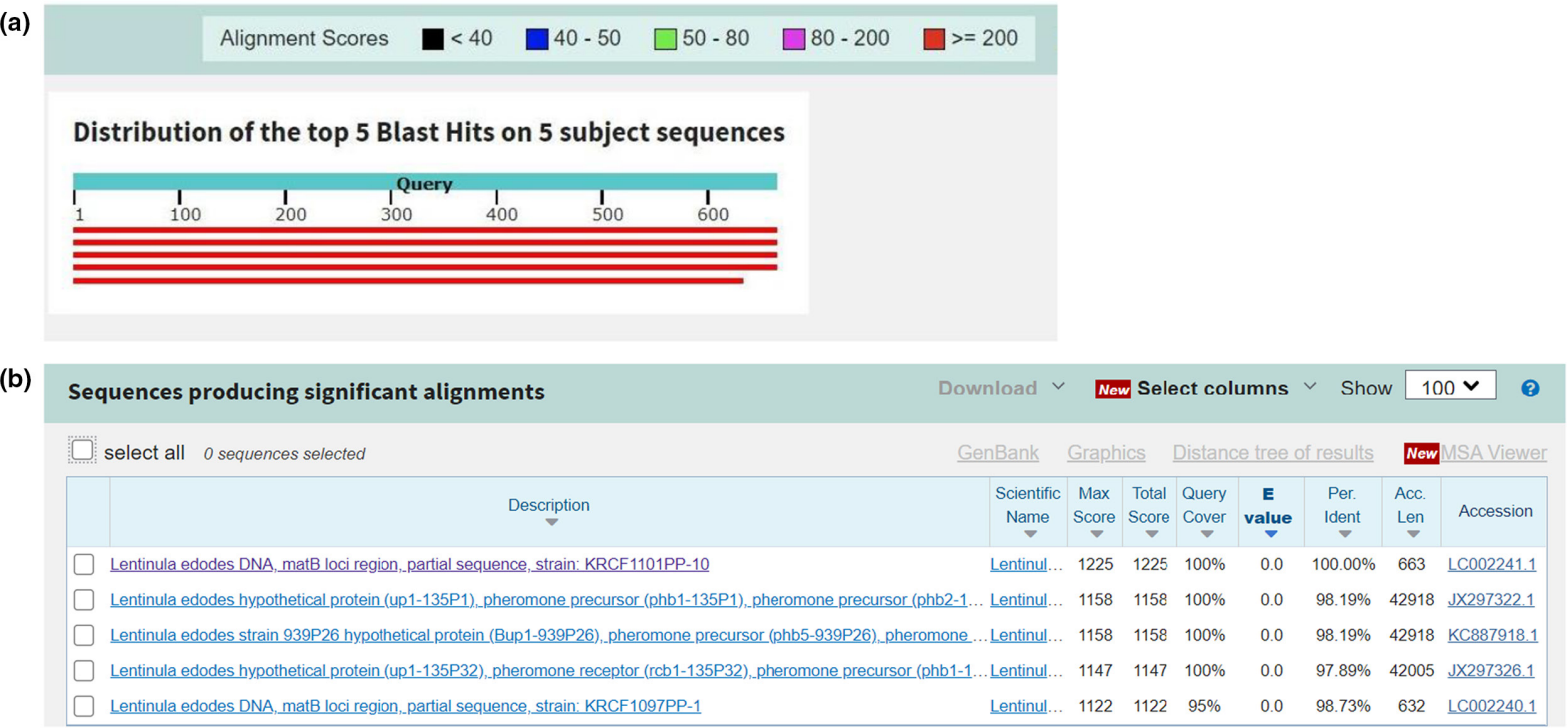
The homology analysis of *matB* gene. The BLAST analysis of *matB* in Nucleotide collection database (a), the detailed information about the BLAST result (b)

The use of endogenous reference genes makes the detection of *L. edodes* more accurate and applicable. Endogenous reference genes must be species specific and have a low and consistent copy number within the same species (Shang et al., 2014). To select suitable endogenous reference genes, we searched the DNA information of *L. edodes* in the NCBI Nucleotide database, collected a large amount of gene information, and screened several candidate genes. BLAST alignment and homology analysis revealed that the *matB* gene (GenBank No. LC002241.1) of *L. edodes* DNA showed no homology with other genes from different varieties of the mushroom. The BLAST result and detailed information of *matB* are provided in Figure 1. Then, Primer Premier 5.0 software was used to design primers for the screened endogenous reference gene *matB* and used for qualitative and quantitative PCR.

### 3.2 DNA‐extraction results

**FIGURE 2:**
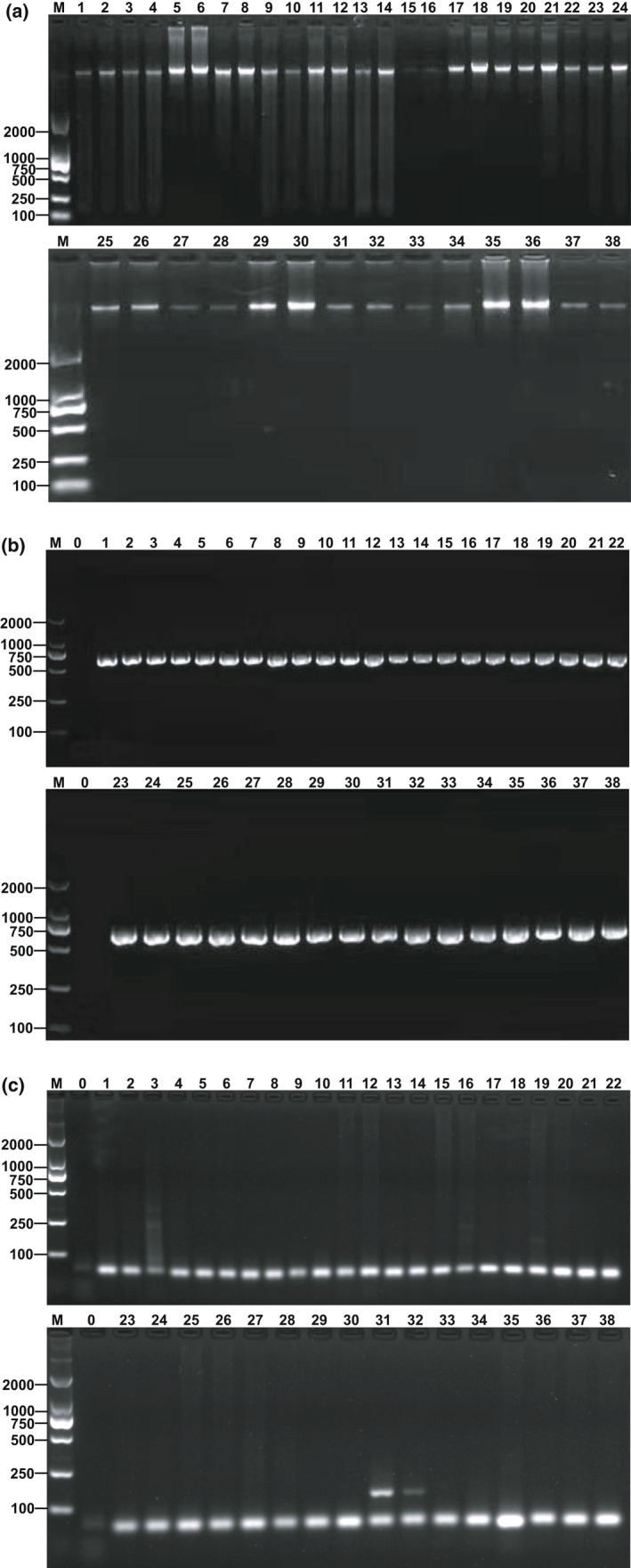
The electrophoresis profile of genomic DNA from the 19 tested species of mushrooms (a), the electrophoresis profile of ITS PCR products (b), the specificity detection results of the qualitative PCR (c), Lane M: DNA Marker DL 2000; 0: negative control; 1 to 2: *Boletus auripes*; 3 to 4: *Pleurotus ostreatus*; 5 to 6: *Boletus brunneissimus*; 7 to 8: *Lactarius volemus*; 9 to 10: *Russula virescens*; 11 to 12: *Thelephora ganbajun*; 13 to 14: *Cantharellus cibarius*; 15 to 16: *Tricholoma matsutake*; 17 to 18: *Boletus rubellus*; 19 to 20: *Boletus griseus*; 21 to 22: *Russula vinosa*; 23 to 24: *Boletus subsplendidus*; 25 to 26: *Termitornyces albuminosus*; 27 to 28: *Catathelasma ventricosum*; 29 to 30: *Ramaria botrytoides*; 31 to 32: *Lentinus edodes*; 33 to 34: *Agrocybe aegerita*; 35 to 36: *Pleurotus eryngii*; 37 to 38: *Tricholoma gambosum*

DNA samples from 19 mushroom species were analyzed using 1% agarose gel (Figure 2a). Results showed that highlighted lanes appeared in each lane, indicating high DNA integrity. PCR products amplified using fungus universal primer ITS were analyzed using 2% agarose gel (Figure 2b). Results showed that all DNA fragments were effectively amplified, and the lanes were clear and neat, indicating that DNA quality was good and could be used for subsequent experiments. Through NanoDrop 2000 measurement, the ideal A260/A280 ratio was between 1.8 and 2.0, indicating high DNA purity. The DNA concentration of *L. edodes* was 25.6 ± 5.6 ng/μl.

### 3.3 Species specificity of the qualitative PCR assays

The *matB*‐F/R primers were used for general PCR amplification to qualitatively verify the interspecific specificity of *L. edodes* endogenous genes. The qualitative results (Figure 2c) showed that *matB* gene was successfully amplified by *L. edodes* genomic DNA with obvious lanes, whereas no amplification products were found in other mushrooms. Results showed that *matB* gene was highly specific to *L. edodes* compared with other mushrooms.

### 3.4 Sensitivity of qualitative and real‐time quantitative PCR assays

**FIGURE 3:**
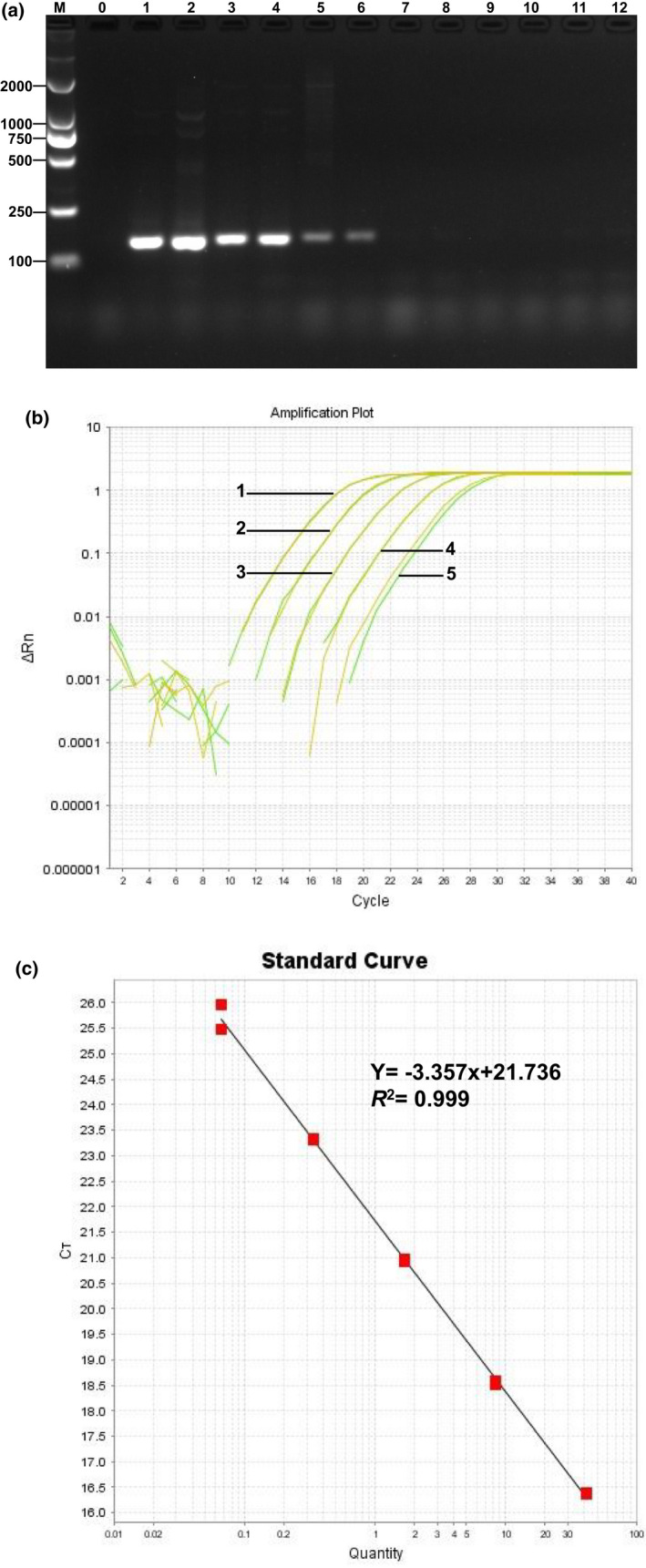
Sensitivity of the *matB* gene detection in qualitative and real‐time quantitative PCR. The sensitivity of the qualitative PCR (a), Lane M: DNA Marker DL 2000; 0: negative control; 1 to 2:25 ng/μl; 3 to 4:5 ng/μl; 5 to 6:1 ng/μl; 7 to 8:0.2 ng/μl; 9 to 10:40 pg/μl; 11 to 12:8 pg/μl. The sensitivity of the quantitative PCR (b), the concentration of DNA in each reaction is: 1:10 ng/µl; 2:2 ng/µl; 3:0.4 ng/µl; 4:80 pg/µl; 5:16 pg/µl. The standard curve of the quantitative PCR (c)

To detect the sensitivity to quantitative PCR, genomic DNA of *L. edodes* was subjected to fivefold serial dilution ranging from 25 ng/μl to 8 pg/μl. As shown in Figure 3a, when the content of the DNA template was 25, 5, and 1 ng/μl, clear specific lanes appeared on the electrophoretic pattern, and the brightness gradually decreased. With decreased content of the DNA template to 0.2 ng/μl, no amplification products were detected. Conventional PCR accommodates detected DNA samples containing as little as 1 ng/µl.

The samples were subjected to fivefold serial dilution ranging from 10 ng/μl to 3.2 pg/μl and then detected by real‐time quantitative PCR to quantitatively determine the detection sensitivity of specific primers of *L. edodes* endogenous genes. As shown in Figure 3b, when the template content was 16 pg/μl, specific amplification still occurred, and the Ct value was significantly higher than that of the negative control, indicating that the detection limit of real‐time quantitative PCR was 16 pg/μl. To ensure the feasibility of the quantitative system in relative quantitative analysis, the standard curve of *matB* was established using the quantitative PCR system. A linear relationship (*R* ^2^ = 0.999, slope = −3.357) was determined between the DNA quantities and Ct values (Figure 3c).

These results further indicated that *matB* gene was suitable to detect *L. edodes*. Moreover, the established *matB* real‐time quantitative PCR system was suitable for the accurate quantitative detection of *L. edodes*.

### 3.5 Application of *matB* as a reference gene to detect processed *L. edodes* products

**FIGURE 4:**
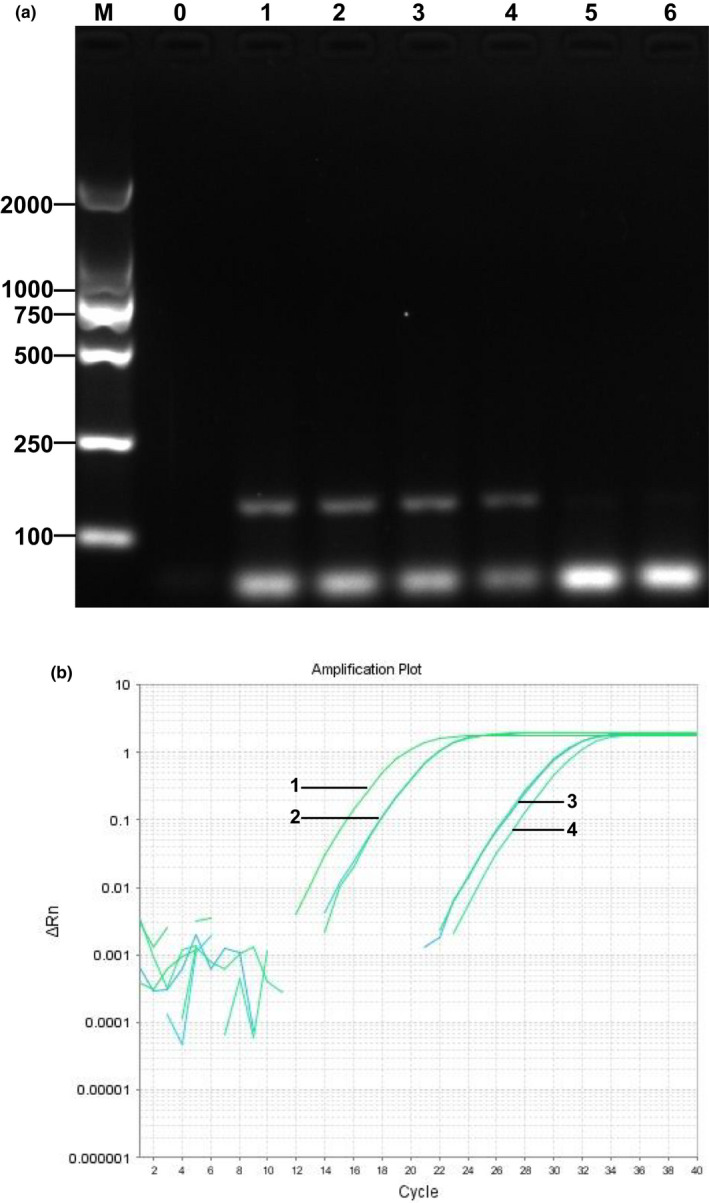
Application of *matB* to detect different processed mushroom products. The qualitative PCR detection (a), Lane M: DNA Marker DL 2000; 0: negative control; 1 to 2: positive control; 3 to 4: *Lentinus edodes* sauce; 5 to 6: *Pleurotus eryngii* sauce. The quantitative PCR detection (b), lane 1: positive control; 2: *Lentinus edodes* sauce; 3: *Pleurotus eryngii* sauce; 4: negative control

We used the established real‐time quantitative PCR system to detect two kinds of *L. edodes* products and confirm the feasibility of establishing the system. To confirm such feasibility, DNA extracted from fresh *L. edodes* was used as a positive test for *L. edodes* sauce and *Pleurotus eryngii* sauce. We amplified the DNA templates extracted from the food samples by using *matB*‐F/R in conventional PCR (Figure 4a). Results demonstrated that the component of *L. edodes* could be detected in *L. edodes* sauce but not in *Pleurotus eryngii* sauce. Results of real‐time quantitative PCR (Figure 4b) were consistent with those in qualitative PCR. The test results were consistent with the food label.

## 4 CONCLUSION

Food adulteration is occurring increasingly often today. Wild edible mushrooms are popular among consumers because of their good flavor and high nutritional value. Due to seasonal limitations and other reasons, adulteration of expensive wild edible mushrooms may occur. In the detection of food adulteration, the PCR amplification of endogenous reference gene is becoming increasingly important. In this paper, specificity and sensitivity verification revealed that *matB* gene can be used as an endogenous reference gene to detect *L. edodes* based on PCR. The specificity and detection limit of *matB* gene was verified by qualitative and real‐time quantitative PCR. The detection limit of real‐time quantitative PCR was as low as 16 pg/μl which indicates this method is sufficiently sensitive. The method can be used to detect processed *L. edodes* products containing small amounts of target genomic DNA. Results showed that the method could be used to detect *L. edodes* processed products with a good detection limit and could be used to detect the adulteration of wild mushrooms. It provides methodological support for the regulation of adulterated wild mushroom products containing the component of *L*. *edodes*.

## CONFLICTS OF INTEREST

The authors declare no conflict of interest.
